# The mitochondrial translation machinery as a therapeutic target in Myc-driven lymphomas

**DOI:** 10.18632/oncotarget.11719

**Published:** 2016-08-31

**Authors:** Aleco D'Andrea, Ilaria Gritti, Paola Nicoli, Marco Giorgio, Mirko Doni, Annalisa Conti, Valerio Bianchi, Lucia Casoli, Arianna Sabò, Alexandre Mironov, Galina V. Beznoussenko, Bruno Amati

**Affiliations:** ^1^ Department of Experimental Oncology, European Institute of Oncology, Milan, Italy; ^2^ Center for Genomic Science of IIT@SEMM, Fondazione Istituto Italiano di Tecnologia, Milan, Italy; ^3^ The Institute of Molecular Oncology of the Italian Foundation for Cancer Research, Milan, Italy; ^4^ Present address: IRCCS San Raffaele, Functional Genomics of Cancer Unit, Division of Experimental Oncology, Milan, Italy; ^5^ Present address: Hubrecht Institute-KNAW & University Medical Center Utrecht, Uppsalalaan, Utrecht, The Netherlands

**Keywords:** lymphoma, mitochondria, mitochondrial translation, Myc, Tigecycline

## Abstract

The oncogenic transcription factor Myc is required for the progression and maintenance of diverse tumors. This has led to the concept that Myc itself, Myc-activated gene products, or associated biological processes might constitute prime targets for cancer therapy. Here, we present an *in vivo* reverse-genetic screen targeting a set of 241 Myc-activated mRNAs in mouse B-cell lymphomas, unraveling a critical role for the mitochondrial ribosomal protein (MRP) Ptcd3 in tumor maintenance. Other MRP-coding genes were also up regulated in Myc-induced lymphoma, pointing to a coordinate activation of the mitochondrial translation machinery. Inhibition of mitochondrial translation with the antibiotic Tigecycline was synthetic-lethal with Myc activation, impaired respiratory activity and tumor cell survival *in vitro*, and significantly extended lifespan in lymphoma-bearing mice. We have thus identified a novel Myc-induced metabolic dependency that can be targeted by common antibiotics, opening new therapeutic perspectives in Myc-overexpressing tumors.

## INTRODUCTION

Tumors driven by deregulated *c-myc* generally show oncogene addiction, being sensitive to suppression or inhibition of the c-Myc protein (hereby, Myc) [1, 2]. Myc is also required in tumors driven by other oncogenic events, such as the activation of Wnt or Ras signaling [3, 4], and is generally activated by these signaling pathways, supporting a general requirement for Myc activity in cancer [5, 6]. Myc is a bHLH-LZ family transcription factor that requires dimerization with the partner protein Max in order to bind DNA and regulate gene expression [5]. When over-expressed, Myc activates and represses large sets of target genes, among which must lie the critical effectors of its oncogenic activity [6-9]. Recently, RNAi-based screens have been employed to identify genes that show synthetic lethality with Myc activation or are required for the progression of Myc-driven tumors [10-14]. However, only a handful of established Myc-target genes were involved so far [12, 15-19]. In particular, no screen systematically addressed the functional requirement of Myc-regulated genes in a given tumor type.

Here, we developed a reverse-genetic shRNA screen aimed at identifying Myc-activated genes required for tumor maintenance. A retroviral shRNA library was designed against 241 Myc-induced mRNAs identified in previous work [8 and unpublished data], and was transduced in murine Eμ-*myc* lymphomas. Following expansion of the lymphomas in recipient animals, genomic DNA was analyzed to identify depleted (or “dropout”) shRNAs imparting a selective disadvantage to tumor cells. One of the genes identified in this manner was *Ptcd3*, which encodes a pentatricopeptide repeat protein incorporated in the small subunit of the mitochondrial ribosome, and is required for efficient translation of mitochondrial mRNAs [20, 21]. Knockdown of *Ptcd3* or other mitochondrial ribosomal proteins (*Mrps5* or *Mrps27*) decreased both expression of mitochondrion-encoded proteins and respiration rates, and was detrimental to Myc-driven lymphomas. Chemical inhibition of mitochondrial translation with the antibiotic Tigecycline showed similar effects *in vitro* and led to rapid tumor clearance *in vivo*, significantly extending the survival of lymphoma-bearing mice. Further analysis in non-transformed mouse B-cells and mammary epithelial cells showed that Tigecycline was not generically toxic to all proliferating cells, but selectively killed cells with supra-physiological Myc levels. Altogether, we conclude that the mitochondrial translation machinery is an important effector of Myc in lymphomagenesis and that Myc overexpression is a primary determinant of Tigecycline toxicity. Our data unmask a genetic basis for the elusive anti-tumoral activity of antibiotics [22-24], providing a rationale for their repurposing against Myc-associated tumors.

## RESULTS

### A reverse-genetic screen reveals a critical role for Ptcd3 in lymphoma maintenance

**Figure 1:**
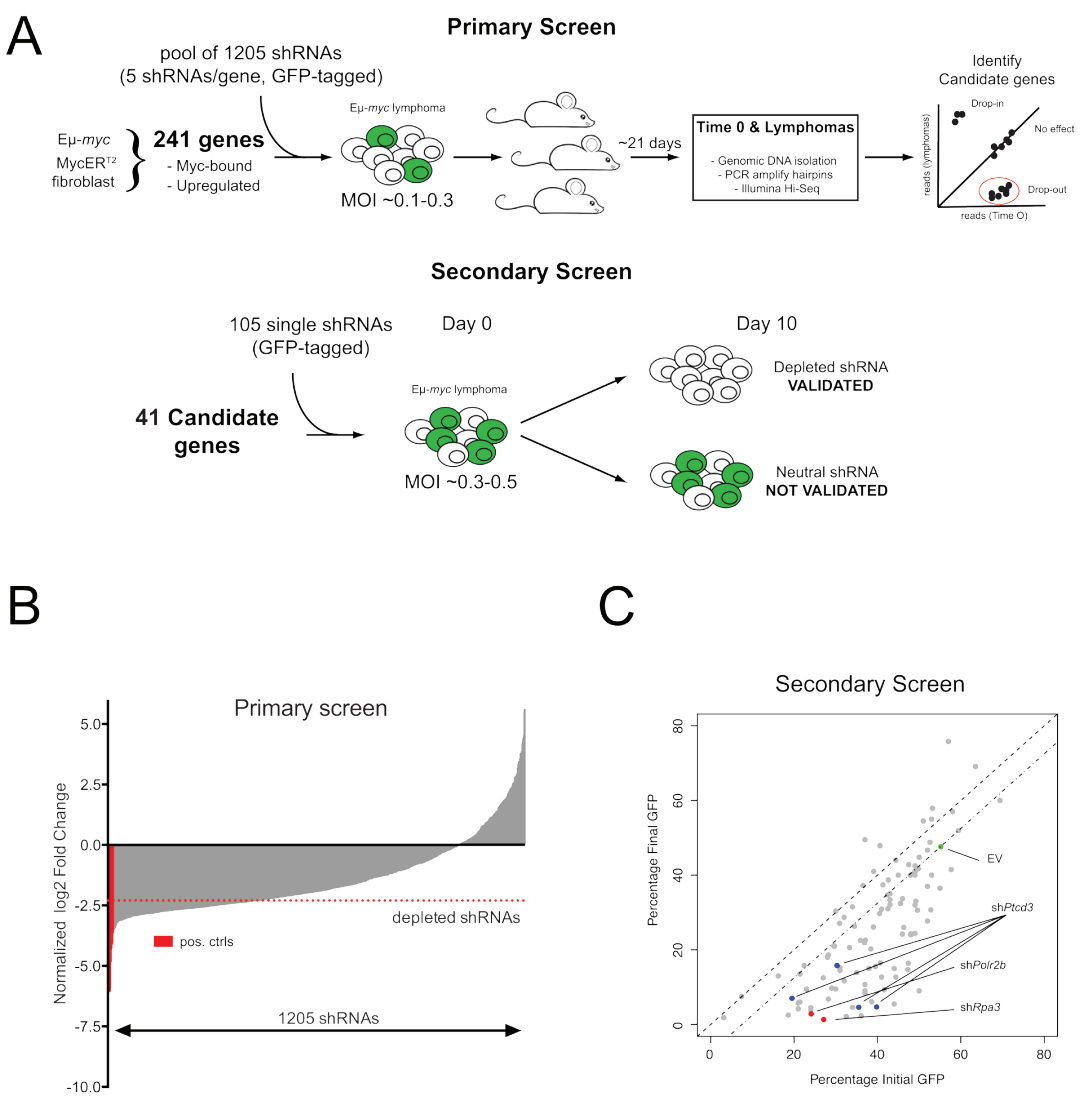
An *in vivo* shRNA screen identifies *Ptcd3* as a critical Myc effector **A.** Schematic of the primary and secondary screens. Eμ-*myc* lymphoma cells were transduced with a pool of 1205 shRNAs and then injected into recipient mice. shRNA representation in lymphomas was assessed by high-throughput sequencing. Candidate genes from the primary screen were further validated in a secondary *in vitro* GFP screen as depicted. **B.** Bar-plot summarizing the results of the primary screen. The average log2 fold change for each shRNA among the four replicates is shown relative to the input population. shRNAs are rank by ascending values. shRNAs targeting known essential genes (*Polr2b, Rpa1 and Rpa3*) are marked in red. A red dotted line indicates the threshold for negative selection (log2FC < −2.3). Candidate genes for the secondary screen were selected based on the number of depleted shRNAs and functional annotation. **C.** Scatter plot summarizing the results of the secondary validation screen. The highlighted dots show cells transduced with the control empty vector (EV, green dot), two shRNAs against essential genes (*Rpa3* and *Polr2b*, red dots) or four different shRNAs against *Ptcd3* (Blue dots). Grey dots show all other shRNAs included in the secondary screen. The percentage of transduced GFP+ cells at Day 0 is reported on the X-axis while the Y-axis shows the fraction of remaining GFP+ cells following 10 days of *in vitro* culture.

We previously mapped Myc-regulated genes during B-cell lymphomagenesis in Eμ-*myc* transgenic mice [8]. Here, we devised a reverse-genetic screen to identify Myc-activated genes that are critical for tumor maintenance (Figure 1A). Analogous to a previous study [10], we constructed a retroviral shRNA library targeting a subset of 241 Myc-induced genes, each mRNA being targeted with 5 different shRNAs (Supplementary Table S1). The library was transduced in quadruplicate into Eμ-*myc* lymphoma cells at low multiplicity of infection to ensure single-copy integration of shRNA constructs in each targeted cell, and each infected population was transplanted into syngeneic recipient mice. After 21 days, the mice were sacrificed and lymphomas collected for DNA extraction. Changes in shRNA representation were assessed by deep sequencing of shRNA guide strands amplified from genomic DNA before and after *in vivo* expansion (Supplementary Table S2). Consistent with previous observations, we observed a general loss of complexity in shRNA distribution following tumor development *in vivo* (Figure 1B) [10, 25]. Nevertheless, control shRNAs targeting essential genes (*Rpa1*, *Rpa3* and *Polr2b*; [26]) were consistently depleted in all four replicates, as were shRNAs targeting genes that either cooperated with Myc (*Prmt5* and *Odc*) [15, 16] or belonged to known oncogenic pathways in Eμ-*myc* lymphomas (*Adsl* and *Rsl24d1*) [18, 19] (Supplementary Table S2). To ensure high-stringency selection criteria, we followed-up only on genes targeted by two or more depleted shRNAs, with at least one shRNA depleted > 5 fold in all four replicates. 41 genes were thus selected for further validation in a secondary *in vitro* competition screen (Figure 1A; Supplementary Table S3), in which we individually tested 2-4 shRNAs per gene: from a total of 105 shRNAs, 78 (74%) conferred competitive disadvantage of infected (GFP^+^) relative to non-infected (GFP^−^) lymphoma cells, validating their initial dropout *in vivo* (Supplementary Table S4; Figure 1C). 27 genes were validated in this manner, with at least two independent shRNAs conferring negative selection in the secondary screen (Supplementary Table S5). Here, we focus on *Ptcd3*, for which 4 independent shRNAs validated in the secondary screen (Figure 1C, blue dots).

### Ptcd3 and other mitochondrial ribosomal proteins are rate-limiting for lymphoma cell proliferation *in vitro*

**Figure 2:**
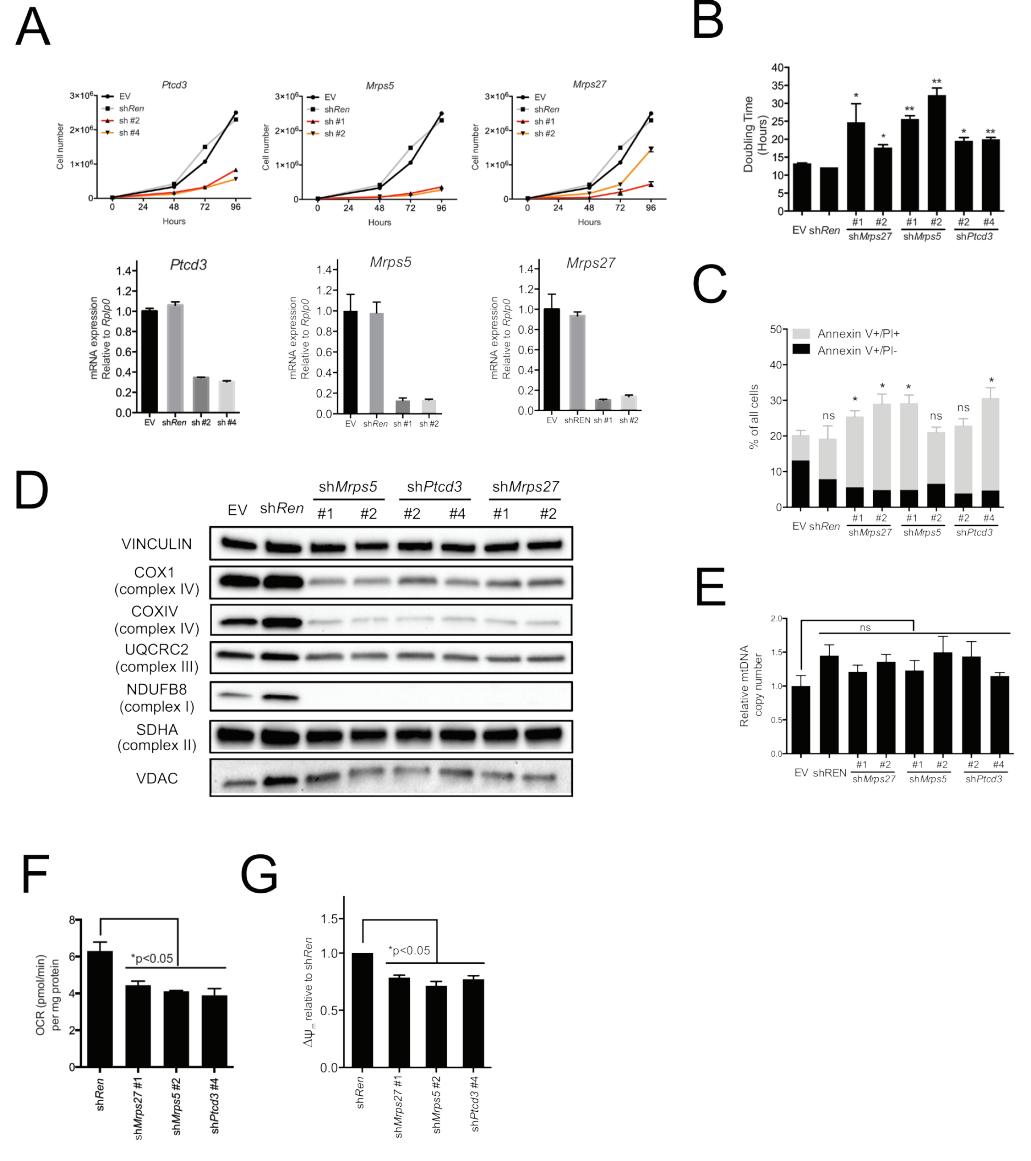
The mitochondrial translation machinery regulates global mitochondrial activity and Eμ*-myc* cell growth **A.** Eμ-*myc* lymphoma cells were transduced with shRNAs against *Ptcd3*, *Mrps5*, *Mrps27*, *Renilla luciferase (Ren)* or a control empty vector (EV). The number of viable cells at each time point (top) was determined by trypan blue staining. For each single shRNA the efficiency of knockdown was evaluated by real-time qPCR three days post-transduction (bottom). Transcript abundance is expressed as mean ± s.d. of triplicate measurements, expressed relative to the EV control and normalized to *Rplp0*. **B.** Doubling time was calculated from the growth curves in A., using the formula described in experimental procedures. **C.** Cell death was evaluated by Annexin V and propidium iodide (PI) staining at the 48hrs time-point. The graph shows the percentage of Annexin V+/PI- (black) and Annexin V+/PI+ cells (grey) corresponding to early and late apoptotic cells, respectively. **D.** Western blot analysis of components of the Electron Transport Chain (ETC) Complexes I-IV following 48hrs of knock-down. As loading controls, we used antibodies against cytoplasmic Vinculin and the mitochondrial Voltage-dependent anion channel (VDAC). **E.** Real-time qPCR quantification of mtDNA, normalized to nuclear DNA. Results are shown as mean ± s.d. from triplicate measurements. **F.** Oxygen consumption rate (OCR) was determined as described in experimental procedures at 48hrs. Data were normalized to total cellular protein contents. **G.** Mitochondrial membrane potential was measured by staining with the cationic cyanine dye DilC1(5) for one representative shRNA per each mitochondrial ribosomal protein (MRP) at 48hrs. Values are expressed relative to the sh*Ren control*. All plotted values are the mean ± s.d. from three independent experiments. * *p* < 0.05; ** *p* < 0.01; ns, not significant, as determined by Student's t test. See also Supplementary Figures S1 and S2.

Pctd3 encodes a pentatricopeptide protein that is incorporated in the 28S subunit of the mitochondrial ribosome and is required for mitochondrial translation [20]. Other genes regulating this process, in particular those encoding Mitochochondrial Ribosomal Proteins (MRPs), were coordinately induced during tumor progression in the Eμ-*myc* model (Supplementary Figure S1 and Supplementary Table S6) [8] pointing to a possible role of the mitochondrial translation machinery for the fitness of Eμ-*myc* lymphoma cells. Consistent with this hypothesis, shRNAs targeting either *Ptcd3, Mrps5* or *Mrps27* (all encoding components of the 28S subunit) impaired proliferation of lymphoma cells (Figure 2A, 2B), delayed progression through all phases of the cell cycle (Supplementary Figure S2A for *Ptcd3*) and caused a slight increase in the number of apoptotic cells, as determined by Annexin-V staining (Figure 2C).

As expected, knockdown of either *Ptcd3, Mrps5 or Mrps27* decreased the levels of COX1 (Figure 2D), a mitochondrion-encoded subunit of the electron transport chain (ETC) Complex IV. The nucleus-encoded proteins NDUFB8, UQCRC2 and COXIV belonging to ETC Complexes I, III and IV, respectively, were also down-regulated, consistent with the destabilization of the whole complexes [27, 28]. Complex II instead contains no mitochondrion-encoded component [29] and its subunit SDHA was unaffected by knockdown of either MRP. In addition, the levels of the outer membrane protein VDAC (Voltage Dependent Anion Channel; Figure 2D) and mitochondrial DNA (mtDNA; Figure 2E) were unchanged, indicating MRP knockdown caused no reduction in global mitochondrial mass.

MRP knockdown also reduced the levels of most of the 13 mitochondrion-encoded RNAs (mtRNAs, Supplementary Figure S2B), indicative of altered mtRNA synthesis and/or turnover [30]. Finally, MRP knockdown led to reduced oxygen consumption rates (OCR) and mitochondrial membrane potential (Figure 2F, 2G). In conclusion, mitochondrial translation is critical for sustained respiratory activity, as well as for the growth and survival of Eμ-*myc* lymphoma cells.

### Pharmacological inhibition of mitochondrial translation phenocopies MRP knockdown

**Figure 3:**
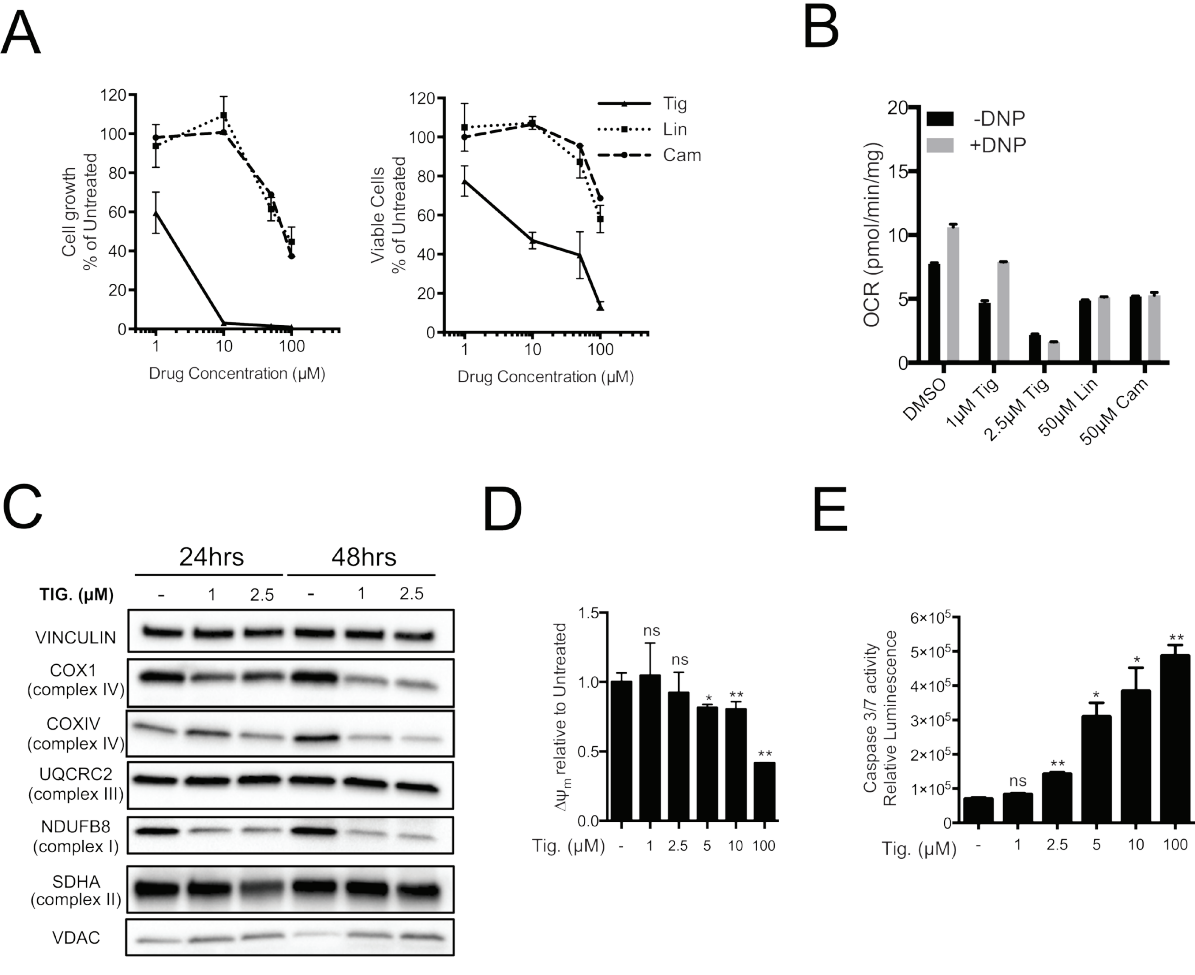
Tigecycline impairs the growth of Eμ*-myc* lymphoma cells and inhibits translation of mitochondrial proteins **A.** Dose-response curve of Eμ-*myc* lymphoma cells treated with the indicated doses of Tigecycline (Tig), Linezolid (Lin) or Cloramphenicol (Cam) for 48hrs. Data are shown as cell numbers (left panel) or viability (right panel), as percentage of untreated cells (mock-treated with DMSO). Cell number and viability was determined by trypan blue staining. **B.** Oxygen consumption rate (OCR) measured as defined in Figure 2F on Eμ-*myc* lymphoma cells treated with the indicated antibiotics or carrier (DMSO). To measure spare respiratory capacity, cells were treated with the ETC uncoupler 2,4-Dinitrophenol (DNP). **C.** Western blot analysis of components of the ETC complexes I-IV. Cells were treated with the indicated concentration of Tigecycline for 24 or 48hrs. The loading controls are Vinculin and VDAC, as in Figure 2D. **D.** Mitochondrial membrane potential was determined by staining with the cationic cyanine dye DilC1(5). **E.** Cleaved caspase 3/7 activity was determined with the caspase 3/7 glo assay luminescent kit. Measurements in D and E were taken after 6 hrs of treatment. Means, s.d. and statistical significance are as defined in Figure 2. See also Supplementary Figures S3 and S4.

Mitochondrial ribosomes are structurally related to bacterial ribosomes and are inhibited by various classes of antibiotics [31-35]. Long-standing observations suggest that cancer cells have augmented sensitivity to these antibiotics [22-24, 36, 37] but the genetic basis for this phenomenon has remained unclear. We thus compared the sensitivity of Eμ-*myc* lymphoma cells to different antibiotics targeting the mitochondrial ribosome, including Tigecycline, Linezolid and Chloramphenicol [23, 34, 38], revealing a dose-dependent inhibition of cell proliferation and viability (Figure 3A). Most importantly, the different anti-proliferative potencies of these antibiotics correlated with their ability to reduce basal mitochondrial respiratory capacity (Figure 3B, black bars) and to prevent increased oxygen consumption by the ETC following uncoupling with 2,4 Dinitrophenol (DNP; grey bars), as expected following inhibition of mitochondrial translation. The strongest effect was observed with Tigecycline, a tetracycline derivative that has also shown effectiveness on cancer cell lines and in pre-clinical models of AML [23, 36, 37].

The dose-dependent effect of Tigecycline was confirmed in the same Eμ-*myc* line (LY27), which is wild type for p53, as well as in an independent p53-null line (LY35) (Supplementary Figure S3A). As seen upon MRP knockdown, Tigecycline treatment suppressed COX1, COXIV and NDUFB8 protein levels, while UQCRC2 and SDHA remained unchanged, VDAC levels were slightly increased and mtDNA abundance remained unaltered (Figure 3C and Supplementary Figure S3B, S3C). The levels of several mtRNAs increased with Tigecycline (*AtpP6*, *CytB*, *Nd2*, *Nd3*; Supplementary Figure S3D), as expected following inhibition of the mitochondrial ribosome [23, 39]. This contrasted with the drop in mtRNA levels seen with MRP knockdown (Supplementary Figure S2B), owing presumably to the disassembly of the small ribosomal subunit - and possibly mtRNA destabilization - only in the latter. Tigecycline also caused depolarization of the mitochondrial membrane and a dose-dependent increase in caspase 3/7 activity (Figure 3D, 3E). Of note, Tigecycline concentrations that already induced cell death (5μM) and reduced respiratory activity (Supplementary Figure S3A; Figure 3C, 3D) did not cause significant decreases in ATP levels (Supplementary Figure S3E), consistent with ATP production through glycolysis (see Discussion). Finally, electron-microscopic analysis revealed alterations in mitochondrial morphology already at the sub-lethal concentration of 1μM, and more markedly at 2.5 μM, with a decrease in the number and length of inner membrane cristae, and occasional perforation of the external membrane (Supplementary Figure S3F).

To confirm that cellular effects of Tigecyline were a consequence of blocking mitochondrial gene expression, we treated Eμ-*myc* lymphoma cells with 2′-C-Methyladenosine (2′CmeA), an inhibitor of the mitochondrial RNA polymerase [40]. The mtRNAs encoding *Mt-CoxIII, Atp6* and *Nd1* were diminished already after 6 hours of treatment, while *CoxIV* mRNA and mtDNA levels remained stable, confirming the selective inhibition of mitochondrial transcription (Supplementary Figure S4A, S4B). As with Tigecycline, increasing doses of 2′CmeA led to decreased growth and viability (Supplementary Figure S4C), and increases in caspase 3/7 activity (Supplementary Figure S4D). These data confirm that mitochondrial-encoded products are critical for the survival of Eμ-*myc* lymphoma cells.

### Tigecycline sensitivity correlates with Myc activation but not with cellular proliferation

**Figure 4:**
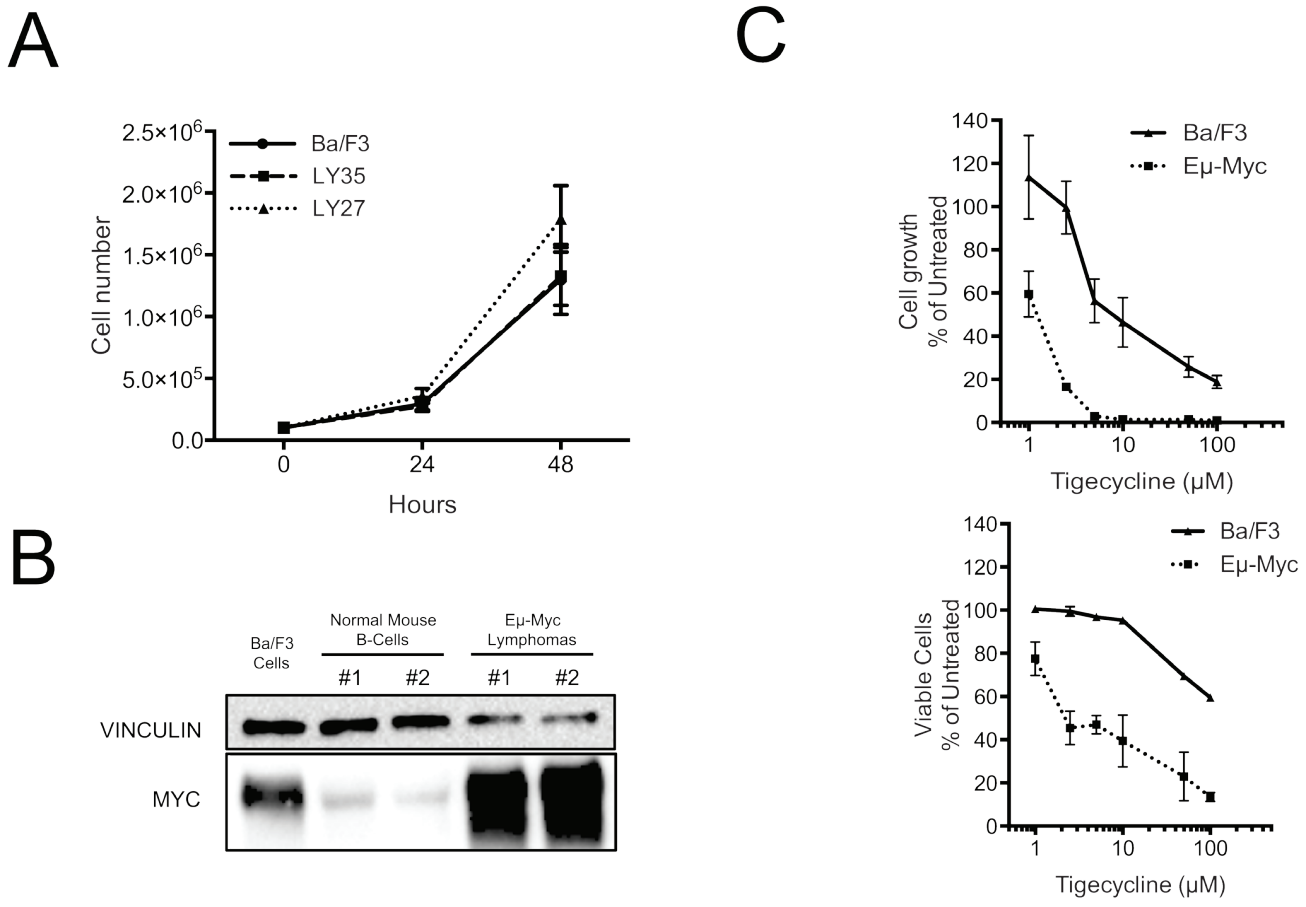
Tigecycline sensitivity is not simply determined by increased proliferation rates **A.** Growth curves for Ba/f3 cells and two different Eμ-myc lymphomas (LY27 and LY35). At each time point, live cells were counted by trypan blue staining **B.** Total proteins extracted from Ba/F3 cells, normal mouse spleen B-cells and Eμ-*myc* lymphomas LY27 (#1) and LY35 (#2) were analyzed by immunoblotting to determine Myc levels. Vinculin was used as a loading control, as in Figure 2D. **C.** Dose-response curve of Eμ-*myc* lymphoma and Ba/F3 cells treated with the indicated doses of Tigecycline, for 48hrs. Total cell numbers (“Cell growth”, upper panel) or viable cells (bottom panel) are expressed as percentage of untreated cells (mock-treated with DMSO). Cell viability was determined by trypan blue staining.

The data presented so far showed that MRP-coding genes are up-regulated during Myc-induced lymphomagenesis and that mitochondrial translation is critical for the fitness of these lymphomas. A main open question at this stage was whether the Tigecycline sensitivity of lymphoma cells is a selective effect of Myc, or merely an indirect consequence of its proliferative activity. To address this question, we initially used the non-transformed B-cell line Ba/F3 [41]. Relative to Eμ-*myc* lymphomas, Ba/F3 cells showed similar proliferation rates (Figure 4A), much lower Myc levels (albeit slightly higher than those of control mouse B-cells: Figure 4B) and significantly lower sensitivity to Tigecycline (Figure 4C). Hence, Tigecycline toxicity correlated with Myc activation in lymphomas, but wasn't a simple consequence of enhanced proliferation.

It is noteworthy here that a previous experiment in the human B-cell line P493-6 was interpreted as a demonstration of Myc-induced sensitivity to Tigecycline, at concentrations of 2.5 μM or higher [23]. However, P493-6 cells are dependent on a Tetracycline-regulated c-*myc* transgene [42]: as predictable based on the structural relatedness of Tetracycline and Tigecycline, and on their ability to regulate tet-dependent reporters [43], as little as 100 nM of either antibiotic was sufficient to ablate Myc expression and suppress proliferation in those cells (Supplementary Figure S5A, S5B). Thus, P493-6 cells cannot be used to address the role of Myc in Tigecycline sensitivity.

**Figure 5:**
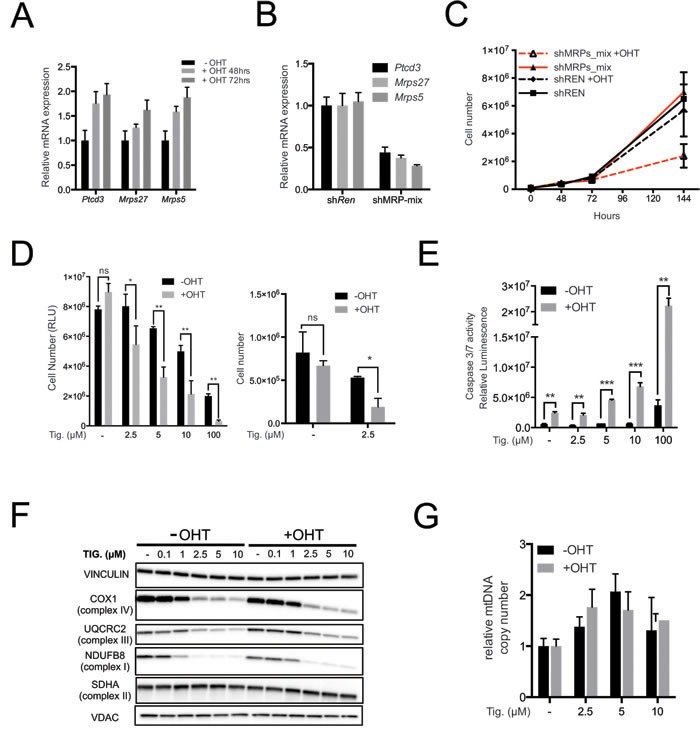
Myc over-expression sensitizes mouse mammary epithelial cells (MMECs) to inhibition of mitochondrial translation **A.** *Ptcd3*, *Mrps27* and *Mrps5* mRNA levels were quantified by RT-PCR in R26-MER^T2^ MMECs treated with 20nM 4-hydroxytamoxifen (OHT) for the indicated time relative to untreated samples. Data were normalized to *Rplp0* expression. **B.** Simultaneous knockdown of *Ptcd3*, *Mrps27* and *Mrps5* in MMECs. The mRNA expression levels for the three MRPs were quantified by RT-PCR following retroviral transduction. Data were normalized to *Rplp0* expression. **C.** MMECs were simultaneously transduced with shRNAs against *Ptcd3*, *Mrps5* and *Mrps27* (2 shRNAs/gene; shMRP-mix) or Renilla luciferase (sh*Ren*). Cells were grown in the absence or presence of 20nM OHT. The number of viable cells at each time point was determined by trypan blue staining. **D.** Relative numbers of R26-MERT^2^ MMECs, as measured with cell titer glo (left panel) or trypan blue exclusion assay (right panel). Before the addition of Tigecycline, cells were pre-treated with 20nM 4-hydroxytamoxifen (+OHT) for 72hrs or left untreated (−OHT). Tigecyline was then added at the indicated doses, with or without 20nM OHT, for an additional 72 hours. **E.** Caspase 3/7 activity was measured as in Figure 3E after 72 hours of treatment with Tigecycline as indicated. The bars represent the absolute luminescence signals in untreated *versus* OHT-treated samples. **F.** Total proteins extracted from MMECs cells were analyzed by immunoblot to determine the expression of components of the ETC Complexes I-IV. The loading controls are Vinculin and VDAC, as in Figure 2D. **G.** RT-PCR quantification of mtDNA, as in Figure 2E. Means, s.d. and statistical significance are as defined in Figure 2, with the addition of *** *p* < 0.001. See also Supplementary Figure S6.

In order to directly address the effect of Myc activation, we used immortalized primary mouse mammary epithelial cells (MMECs) derived from the mammary glands of 6-8 weeks old R26-MER^T2^ mice [44] that constitutively express the 4-hydroxy-tamoxifen (OHT)-activatable MycER^TAM^ fusion protein [45]. The *Mrps5*, *Mrps27* and *Ptcd3* mRNAs were up-regulated following OHT stimulation (Figure 5A). These mRNAs were targeted concomitantly by infecting R26-MER^T2^ MMECs with a pool of retroviral shRNA vectors (shMRP-mix: 2 per gene), achieving ~60-70% reduction of each mRNA (Figure 5B). Neither shMRP-mix, nor OHT alone affected R26-MER^T2^ cultures, but the two treatments together resulted in a marked decrease in cell proliferation (Figure 5C). In a similar manner, OHT treatment sensitized R26-MER^T2^ MMECs to increasing doses of Tigecycline (Figure 5D), with a strong cooperative effect in the induction of apoptosis (Figure 5E). As seen in the Eμ-*myc* model, Tigecycline treatment caused down-regulation of both mitochondrion- (COX1) and nucleus-encoded ETC subunits (UQCRC2 and NDUFB8) while SDHA was not significantly altered (Figure 5F) and mtDNA slightly increased (Figure 5G). Most importantly, R26-MER^T2^ MMECs showed similar proliferation rates in the presence or absence of OHT (Figure 5C). Hence, MycER^TAM^ activation sensitized cells to either MRP knockdown or Tigecycline, an effect that could not simply be accounted for by the proliferative status of the cells.

The above data indicate that Myc activation augments the dependency of cells upon mitochondrial translation. On this basis, one might predict a similar effect on mitochondrial transcription. Indeed, OHT treatment also sensitized R26-MER^T2^ MMEC cells to 2′CmeA (Supplementary Figure S6A, S6B). As expected, mtRNAs were depleted upon 2′CmeA treatment regardless of OHT, while mtDNA was not and expression of the nucleus-encoded *Cox-IV* mRNA was unchanged (Supplementary Figure S6C, S6D). Thus, as also demonstrated by Oran et al. in the accompanying paper [46], Myc activation sensitized cells to inhibition of mitochondrial transcription.

Taken together, Myc enhances the cell's dependency upon mitochondrial biosynthetic activity, whether at the transcriptional or translational level.

### Tigecycline reduces tumor burden and significantly extends survival of lymphoma-bearing mice

To address the significance of our findings in a pre-clinical setting, we tested whether Tigecycline was effective in the treatment of Eμ-*myc* lymphomas *in vivo*. Consistent with previous observations [23], we did not observe any macroscopic sign of toxicity or weight loss upon bi-daily intraperitoneal injection of 100mg/kg Tigecycline for 2 weeks (Supplementary Figure S7A). Histological staining of spleen, liver, kidney and heart showed no evident abnormalities in treated animals, relative to untreated controls (Supplementary Figure S7B). Cell counts in either peripheral blood or spleen revealed modest changes - all below statistical significance - in the numbers of circulating white blood cells (WBC), platelets, or B-cells (Supplementary Figure S7C, S7D). We thus maintained the same treatment protocol in further experiments.

**Figure 6:**
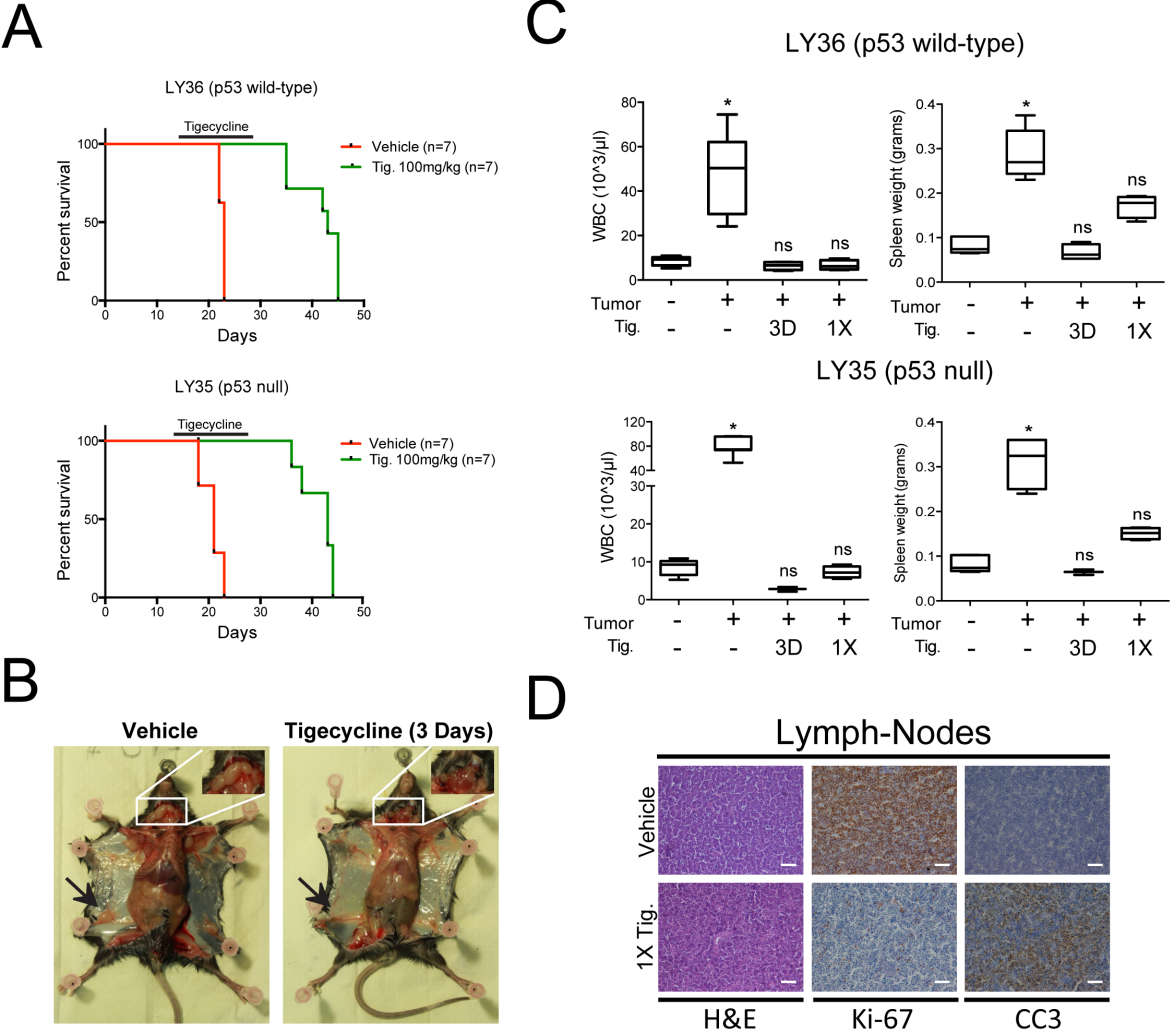
Inhibition of mitochondrial translation with Tigecycline provides a therapeutic window in a mouse model of Myc-induced lymphomas **A.** Kaplan-Meier survival curves of Eμ-*myc* lymphoma-transplanted mice treated with 100 mg/kg Tigecycline or vehicle (saline solution). 5*10^5^ cells of either a p53 wt (LY36) or null (LY35) lymphoma were injected intravenously into C57Bl/6 recipient mice. Treatment with Tigecycline was started on day 14, when tumors were already palpable. Tigecycline was injected *via* intraperitoneal injection twice a day for 14 days. *p* < 0.001 for treated relative to untreated for both lymphomas, as determined by Log-rank (Mantel-Cox) test. **B.** Representative images of LY36 mice treated with vehicle or Tigecycline for three consecutive days. Note the absence of enlarged inguinal (black arrows) and cervical (white inset) lymph-nodes in Tigecycline-treated, as compared with vehicle-treated mice. **C.** Total white blood cell (WBC) count and spleen weight in vehicle and Tigecycline-treated mice after a single Tigecycline administration (1X) or 3 days of treatment (3D). Healthy C57Bl/6 mice were used as controls. Means, s.d. and statistical significance are as defined in Figure 2. **D.** Immunohistochemical stainings of lymph-nodes from mice treated with a single dose of tigecyline or vehicle. Representative H&E, Ki-67 and cleaved caspase 3 (CC3) images at 10X magnification are shown. Scale Bar 100 μm. See also Supplementary Figure S7.

Eμ-*myc* lymphomas can be readily transplanted into syngeneic recipient mice and give rise to aggressive, disseminated tumors within 2-3 weeks. We thus injected 5*10^5^ tumor cells derived from primary lymphomas into the tail-vein of C57BL/6J mice and started Tigecycline treatment after 14 days, when tumors were palpable. Independently from the p53 status of the tumor, Tigecycline significantly extended survival of the recipient animals (Figure 6A). By day three, treated mice showed no signs of disease, were as active as control healthy mice (data not shown), and showed reduced lymph-node infiltration, normalized circulating WBC counts and spleen weight (Figure 6B, 6C). The latter parameters were significantly normalized already 24 hours after a single injection of Tigecycline (1X, Figure 6C), associated with disappearance of Ki-67 and induction of cleaved caspase 3 (CC3) staining in lymph-nodes (Figure 6D), indicating a fast transition from a proliferative to an apoptotic state. Following cessation of the treatment, mice eventually succumbed from lymphoma, indicating that Tigecycline alone was insufficient for full disease eradication.

### Tigecycline treatment impairs the growth of Burkitt's Lymphoma xenografts

**Figure 7:**
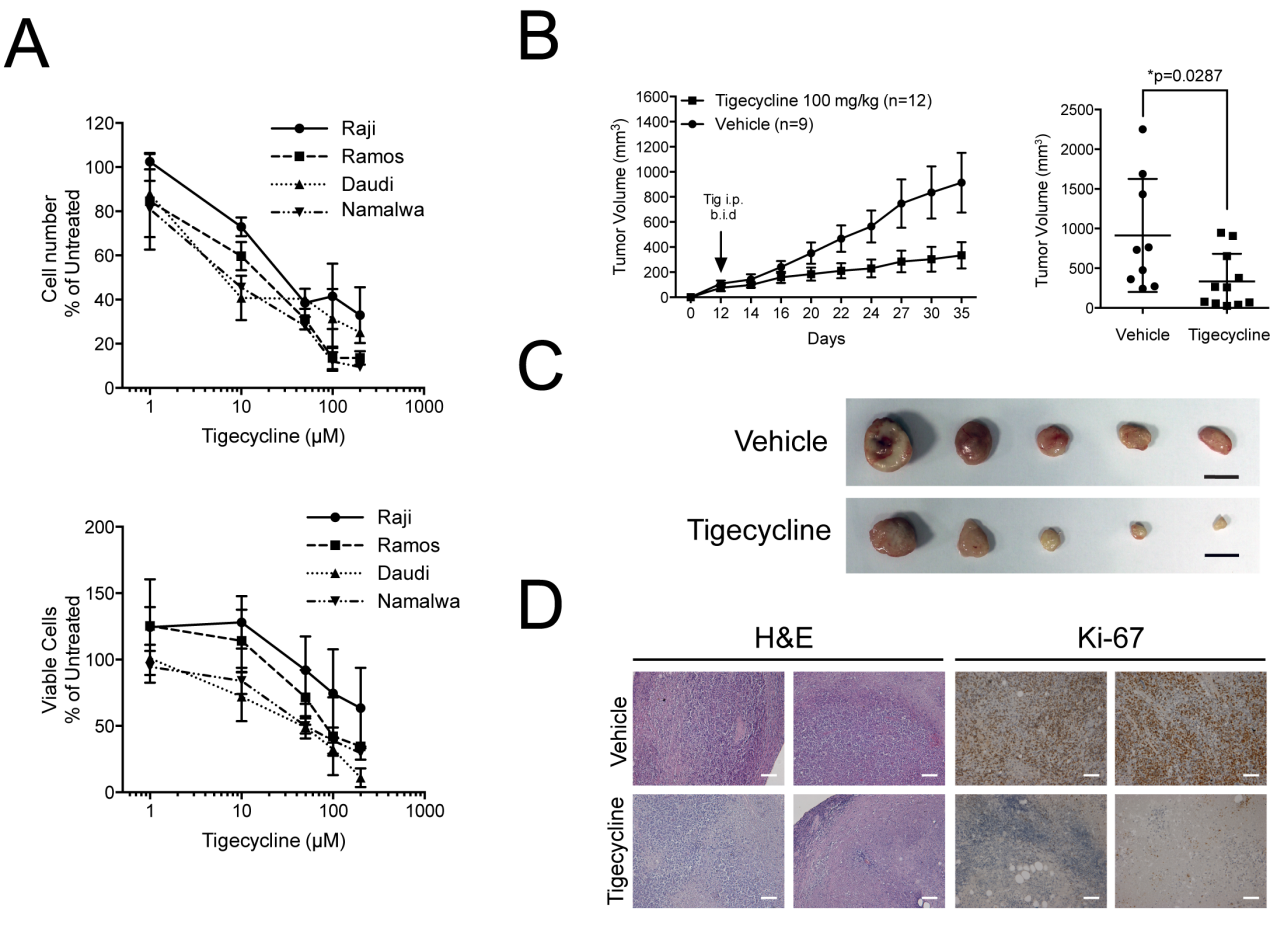
Tigecycline shows activity against human Burkitt's lymphoma xenografts **A.** Relative cell viability (top) and cell numbers (bottom) for four Burkitt's lymphoma cell lines treated with the indicated doses of Tigecycline for 48 hrs. Values are represented as in Figure 3D. **B.** Left: Raji cells were injected subcutaneously into the flank of nude mice. Starting at day 12, when tumors were measurable, mice were injected intra-peritoneally (i.p.) twice per day (b.i.d.) with either 100 mg/kg Tigecycline or vehicle control. Tumor volume was assessed using a caliper at the indicated days. At day 35, mice were sacrificed and tumors collected for further analysis. Right: mean tumor volume at day 35 in vehicle *versus* Tigecycline-treated mice. Values are shown as the mean ± standard error of the mean (s.e.m.). * *p* < 0.05, as determined by student's *t*-test. **C.** Representative images of subcutaneous tumors from vehicle or tigecyline-treated mice at day 35. Scale bar: 1cm. **D.** Representative H&E- and Ki-67-stained sections from vehicle or tigecyline-treated mice at 10X magnification. Scale Bar: 100 μm.

Based on the above data, we hypothesized that human Burkitt's Lymphoma (BL) might be sensitive to inhibition of mitochondrial translation. Indeed, the growth of four different BL cell lines was suppressed by Tigecycline with IC50 values ranging from 8.6 to 44 μM (Figure 7A). We then xenografted the least sensitive BL cell line (Raji), let tumors develop and treated the animals with Tigecycline for 14 days, revealing a significant delay - in some cases even a full arrest - in tumor growth relative to vehicle-treated tumors (Figure 7B). Tigecycline-treated tumors were smaller in size, had a fibrotic appearance and lost Ki-67 expression (Figure 7C, 7D). Thus, as Eμ-*myc* lymphomas, BL-derived lymphomas showed a response to Tigecycline in recipient mice.

## DISCUSSION

Thorough mapping of the transcriptional programs regulated by Myc in diverse tumor types will be key to our understanding of its oncogenic activity [6, 7]. However, if we are to discriminate between incidental and critical regulatory events, profiling studies will need to be complemented with functional screens in the relevant tumor models. We previously profiled Myc-regulated transcription during lymphoma progression in Eμ-*myc* transgenic mice and following MycER activation in proliferating fibroblasts [8]. Here, we selected 241 genes induced in both model systems and subjected these to a reverse-genetic screen in lymphomas, uncovering 27 genes whose knockdown imparts a selective disadvantage to tumor cells *in vivo*. Among these was *Ptcd3*, which encodes a Mitochondrial Ribosomal Protein (MRP) [20]. Further analysis revealed that most of the nuclear genes linked to the mitochondrial translation apparatus, and in particular other MRP-coding genes, were activated in a concerted manner in lymphomagenesis, extending previous reports on the regulation of these genes by Myc [47]. Genetic or pharmacological inhibition of mitochondrial translation - the latter with the Tetracycline-related antibiotic Tigecycline - was detrimental to lymphoma cells *in vitro*. Further analysis in non-transformed mouse B-cells and mammary epithelial cells showed that Myc activation sensitized the cells to Tigecycline independently from any effect on cell proliferation. Finally, Tigecycline significantly retarded the progression of either mouse or human lymphomas in recipient mice, providing a therapeutic window in this pre-clinical setting.

We shall remark here that a previous attempt was made to address the response of Myc-overexpressing cells to Tigecycline by using the B-cell line P493-6 [23]. However, these cells are strictly dependent upon a Tetracycline-repressible c-*myc* transgene [42], which as shown here is also potently repressed by Tigecycline, rendering these experiments inconclusive. Thus, prior to our work, no other data had addressed the role of Myc in the sensitivity of tumor cells to Tigecycline. In line with our findings, however, inhibition of human mitochondrial peptide deformylase (HsPDF) blocked mitochondrial translation and induced apoptosis in a Myc-dependent manner in either P493-6 or Burkitt's lymphoma cells [48].

Oncogene-mediated metabolic reprogramming is considered a hallmark of cancer [49, 50]. Myc in particular, has been connected to the Warburg effect, in which cells switch from oxidative phosphorylation to aerobic glycolysis for ATP production [49, 51, 52]. Myc further modulates mitochondrial metabolism and biogenesis, in particular through the regulation of nuclear genes encoding mitochondrial proteins, either directly or through other factors such as PGC1ß and p32/C1QBP [47, 52]. Our data and those of Oran et al. [46] add a new essential link between Myc and mitochondria, showing that augmented expression of the mitochondrial genome - indirectly controlled by Myc itself - is critical for the fitness of Myc-driven tumor cells.

The mechanisms through which Myc sensitizes cells to inhibition of the mitochondrial biosynthetic apparatus remain to be unraveled. As the mitochondrial genome (or mtDNA) encodes 13 subunits of the ETC [53], the effect must be linked to electron transport. Increased production of reactive oxygen species (ROS) by the ETC and the resulting DNA damage have been proposed to mediate the cytotoxicity of other antibiotics (ciprofloxacin, ampicillin and kanamycin) [54]. Oran et al. [46] observed that antioxidants partially rescue apoptosis upon treatment with either 2′CmeA or Tigecycline. However, unlike other antibiotics, neither tetracycline, nor Tigecycline appear to induce ROS directly [23, 54]. Hence, although they may contribute to overall cytotoxicity, ROS are unlikely to be direct effectors of Tigecycline in this setting.

ETC complexes also determine the electrochemical gradient required for the phosphorylation of ADP. Oxidative phosphorylation is indeed a major source of ATP in Myc-overexpressing cells [55, 56], although these cells may efficiently generate ATP through aerobic glycolysis [57, 58]. Consistent with the latter, we observed Tigecycline-induced cell death without decreased ATP levels. Thus, other functions of the ETC might conceivably contribute to Tigecycline sensitivity.

Besides ATP production, the mitochondrial electrochemical gradient regulates membrane permeability and protein import, which in turn control various biosynthetic pathways, including those for lipid precursors, nucleotides, amino acids and metalloproteins, with important consequences for cell growth and division [59-62]. ETC activity also regenerates NAD+ to fuel the biochemical reactions of the TCA cycle, and Myc overexpression in cancer cells has been proposed to redirect TCA intermediates from ATP production to the aforementioned biosynthetic pathways [52, 63]. Therefore, by suppressing the synthesis of mitochondrially-encoded ETC components, Tigecycline may limit the ability of Myc-overexpressing cells to derive essential macromolecular precursors.

Therapies based on inhibition of mitochondrial activities have been proposed for the treatment of human cancers [64, 65]. Since the 1980′s, numerous studies have shown that Tigecycline and other antibiotics are toxic to tumor cells, either alone or in combination with chemotherapy [22-24], although the genetic basis for this effect had remained unclear. Our data imply that activation of c-*myc* is one of the oncogenic lesions sensitizing tumors cells to the inhibition of mitochondrial translation. We also show that Tigecycline acts independently from p53, which is frequently mutated in Myc-driven hematological cancers [66, 67].

In conclusion, the identification of Myc as a determinant of Tigecycline sensitivity provides a new potential indicator for the re-purposing of this antibiotic in the clinic. Further investigations will assess whether this may be extended to other Myc-dependent tumor types and whether Tigecycline may show synergy with current therapeutic agents.

## MATERIALS AND METHODS

### Cell lines and cultures

Eμ*-myc* lymphoma cells were derived from tumor lymph-nodes of either Eμ-*myc* or Eμ-*myc*; p53^+/−^ mice and maintained *in vitro* in a 50:50 mixture of DMEM (Dulbecco modified Eagle medium) and IMDM (Iscove's modified Dulbecco's medium), 10% FBS, 2mM L-glutamine, 1% penicillin/streptomycin, 50 μM ß-mercaptoethanol and non-essential amino acids (NEAA). The status or p53-proficient lymphomas was confirmed by immunoblot analysis of p53 accumulation upon Adriamycin treatment. All experiments were performed in triplicates on a p53 wild-type (LY27) and a p53 null (LY35) lymphoma. Unless otherwise specified all experiments shown in main and supplementary figures were performed on LY27. Mammary epithelial cells (MMECs) were derived from the mammary gland of 6 weeks old Rosa26-MycER knock-in mice (R26-MER^T2^) [44] and were immortalized by simultaneous knock-down of p53 and pRb [68]. Cells were maintained *in vitro* in a 50:50 mixture of DMEM and F12 media supplemented with 5% FBS, 2mM L-glutamine, 1% penicillin/streptomycin, 20ng/mL hEGF, 4μg/μL Heparin, 50ng/mL cholera toxin, 0.5 μg/mL hydrocortisone, 5μg/mL insulin, 10mM HEPES. Ba/F3 cells were mantained *in vitro* in RPMI-1640 medium supplemented with 10% FBS, 2mM L-glutamine, 1% penicillin/streptomycin, 5% WEHI-3B conditioned medium (as a source of IL-3). Burkitt's lymphoma cell lines were cultured in RPMI 1640 (Raji, Daudi and Namalwa) or MEM (Ramos) supplemented with 10% FBS, 2mM L-glutamine, 1% penicillin/streptomycin, 1mM Sodium Pyruvate, 10mM HEPES. All cells were incubated at 37°C in a humidified air atmosphere supplemented with 5% CO2.

For drug treatment experiments Tigecycline (Sequoia Research Products) and 2′-C-Methyladenosine (Santa Cruz) were dissolved in DMSO. When needed, dilution were prepared fresh from stock solution in DMSO and added directly to the culture medium. Ethidium Bromide (Sigma) was diluted in water and added to the culture medium at a final concentration of 50ng/ml.

### shRNA library screen

To generate the shRNA library, we selected 241 candidate genes that were induced in Eμ-*myc* lymphomas and MycER-expressing fibroblasts [8 and unpublished data] and cloned them into the miR30-based LMS vector. See supplementary methods for a detailed explanation of the procedure.

### Immunoblot analysis and antibodies

5 to 10×10^6^ cells were lysed with RIPA buffer (50mM Tris-cl pH7.6, 1%NP40, 1% deoxycholic acid, 0.1% SDS, 1mM EDTA, 150mM NaCl) supplemented with protease and phosphatase inhibitors (Roche), sonicated in a water bath sonicator and cleared by centrifugation at 13000xg. 30 μg of cleared lysates were electrophoresed on SDS-PAGE gels and immunoblotted with the following primary antibodies: mouse monoclonal anti-Complex I subunit NDUFB8 (Invitrogen, cat. #459210); mouse monoclonal anti-Complex III subunit UQCRC2 (Abcam, cat. #ab14745); rabbit polyclonal anti-VDAC (Pierce, cat. #PA1-954A); mouse monoclonal anti-MT-COI (MitoScience, cat. #1D6E1A8); mouse monoclonal anti-SDHA (Invitrogen, cat. #459200); mouse monoclonal anti COX IV (Abcam, cat. #ab33985); rabbit monoclonal anti-c-Myc Y69 (Abcam, cat. #GR144732-28); mouse monoclonal anti-vinculin (Abcam, cat. #ab18058). After incubation of the membranes with appropriate secondary antibodies, chemiluminescent detection was done through a CCD camera using the ChemiDoc System (Bio-Rad). Quantification of protein levels was done using the Image Lab software.

### RNA extraction and analysis

Total RNA was purified using the Maxwell LEV simplyRNA Purification Kit (Promega), according to the manufacturer's instructions. Complementary DNA (cDNA) was produced using the reverse-transcriptase ImPromII (Promega). A total of 10 ng of cDNA was used for real-time PCR reactions with FAST SYBR Green Master Mix (Applied Biosystems). See Table S7 for the full list of primers.

### Quantification of mitochondrial DNA

Quantification of mtDNA was performed with real-time PCR reactions using FAST SYBR Green Master Mix (Applied Biosystems) and 50ng of total DNA. The following primers were used: CoxI_fw (5′- TCG GAG CCC CAG ATA TAG CA −3′) and CoxI_rv (5′ -TTT CCG GCT AGA GGT GGG TA −3′). Data were normalized to the amount of nuclear DNA as measured with the following primers: Rplp0_fw (5′ -TTC ATT GTG GGA GCA GAC- 3′) and Rplp0_rvCAG CAG TTT CTC CAG AGC- 3′).

### Cell growth and apoptosis assays

In all experiments, cell growth was assessed by either trypan blue exclusion with an automated cell counter (TC20™, Bio-rad) or the CellTiter-Glo^®^ Luminescent Cell Viability Assay (Promega), following manufacturer's instructions. Doubling time (DT) was calculated from the the growth curves using the formula DT = T ln2/ln(X_e_/X_b_), where T = time interval, X_e_ = final number of cells, X_b_ = initial number of cells.

Caspase activity was evaluated with the Caspase-Glo^®^ 3/7 Assay (Promega), following manufacturer's instructions. For AnnexinV staining, 5×10^5^ cells were washed once with Annexin buffer (10mM HEPES, 140mM NaCl, 2.5mM CaCl_2_) and stained in 50 μL annexin buffer plus AnnexinV-APC antibody (BD biosciences), for 1 hour at RT in the dark. Cells were then washed with Annexin buffer and resuspended in 500μL PBS+50μg/mL PI. The fluorescent signal was acquired immediately after staining on a FACSCalibur flow cytometer (BD biosciences).

### Oxygen consumption rate, ATP and mitochondrial membrane potential measurement

Oxygen consumption of cultivated cells was determined by polarographic analysis using a Clark type Hansateck Oxygraph in 1 ml chamber. Basal respiration rate was determined for 1×10^7^ cells resuspended in fresh medium. For data normalization protein concentration was measured using the DC protein assay (Biorad). ATP was measured from equal amount of cells with the CellTiter-Glo^®^ Luminescent Cell Viability Assay (Promega), following manufacturer's instructions. Mitochondrial membrane potential was determined by staining with the cationic cyanine dye DilC1(5) (Invitrogen). Briefly, cells were treated with 50 nM DiIC1(5) in PBS and incubated at 37°C, 5% CO2, for 20 minutes. Cells were then washed once with PBS, resuspended in 500 μL PBS, and fluorescence was acquired immediately analyzed on the FACSCalibur flow cytometer (BD biosciences) with 633 nm excitation laser.

### Histology and immunohistochemical staining

Samples for histological analysis were fixed in 4%PFA overnight at 4°C immediately after collection from the animals. For immunohistochemistry analysis, 5 μm sections were de-waxed and re-hydrated through an ethanol scale, heated in citrate solution (BioGenex #HK086-9K) in a water bath at 99°C for 30 minutes for antigen unmasking, washed once in water and treated with 3% H_2_O_2_ for quenching of endogenous peroxidases. Slides were incubated with antibody against Ki-67 (#M7249 Dako, 1:500) or Cleaved Caspase-3 (#9661 Cell Signaling, 1:200), in a blocking solution (2% BSA, 2% goat serum, 0.02% Tween20, in TBS 1x) for 3h at RT. After primary incubation, slides were washed twice with TBS 1x, and incubate with the secondary antibody (DAKO Cytomation Envision System Labelled Polymer-HRP) for 45 minutes. After 2 washes with TBS, the signal was revealed with a DAB peroxidase substrate solution (DAKO) for 2-10 minutes. Slides were finally counterstained with hematoxylin, de-hydrated through alcoholic scale and mounted with Eukitt (Bio-Optica).

### Assessment of tigecycline activity in mouse lymphomas models

Experiments involving animals have been done in accordance with the Italian Laws (D.lgs. 26/2014), which enforces Dir. 2010/63/EU (Directive 2010/63/EU of the European Parliament and of the Council of 22 September 2010 on the protection of animals used for scientific purposes). See supplementary methods for a detailed explanation of the experimental procedures.

### Statistical analysis

All data are expressed as mean and standard deviation (s.d.) or standard error of the mean (s.e.m.) to account for variation among experimental measures. Statistical analyses were performed by unpaired two-tailed Student's t test. Differences were considered statistically significant at *p* < 0.05. Statistical evaluation of survival curves was performed using the logrank (Mantel-Cox) test.

## SUPPLEMENTARY MATERIALS TABLES AND FIGURES




