# Supplementary material for: The mitochondrial translation machinery as a therapeutic target in Myc-driven lymphomas
Source: Oncotarget. 2016 Aug 31;7(45):72415–30. doi: 10.18632/oncotarget.11719 (PMC5341918; doi:10.18632/oncotarget.11719)
Supplement: Supplementary file 1 [file oncotarget-07-72415-s001.pdf]

# The mitochondrial translation machinery as a therapeutic target in Myc-driven lymphomas

**Supplementary Material**

**shRNA library screen**

***- Vectors and Library construction***

Using an online tool (http://euphrates.mit.edu/cgi-bin/shRNA/index.pl), we designed five independent miR-30-based shRNA oligonucleotides per gene (length: 97 bp), yielding a total of 1205 oligonucleotides targeting 241 different genes that were synthesized on a custom 4K solid array (LC Sciences, Houston, USA). This pool was used as template in a PCR reaction using the following primers: XhoI_fw (5'- CAG AAG GCT CGA GAA GGT ATA TTG CTG TTG ACA GTG AGC G-3') and EcoRI_rv (5'- CTA AAG TAG CCC CTT GAA TTC CGA GGC AGT AGG CA-3'). Amplified PCR products were digested with XhoI/EcoRI and cloned downstream of the MSCV promoter in the mir-30 based retroviral vector LMS, which expresses GFP (Dickins et al., 2005). 5 single shRNAs targeting known essential genes (*Rpa1*, *Rpa3*, *Polr2b*) were manually added to the library of shRNAs

***- Lymphoma infection and transplant***

Eμ-*myc* lymphomas were infected at low multiplicity of infection (MOI = 0.1 to 0.2) in order to ensure that each infected cell carried no more than one viral insert. 48hrs later, $1x10^6$ cells were transplanted by tail-vein injection into syngeneic C57BL/6 females for in vivo expansion. Genomic DNA was used to determined shRNA distributions in the tumor populations by Illumina sequencing and depleted shRNAs (see below) selected for the *in vitro* secondary validation screen. In the secondary screen viral preparations were titrated in order to achieve a transduction efficiency of ~10 % to ~60%, and the fraction of $GFP^+$ cells was measured 2 d after infection (day 0) and again after 10 day of culture.

***- Tumor sequencing and Analysis***

For evaluating the shRNA distributions in tumor cell populations before and after passage *in vivo*, we designed a PCR-based barcoding strategy. Briefly, three different lymphomas were collected and processed from each mouse. DNA was extracted from

lymphoma cell pellets using the Gentra Puregene kit (Qiagen). shRNA sequences integrated into genomic DNA were recovered by PCR amplification with primers annealing to the shRNA loop (forward primer) and the miR30 region (reverse primer). The primers also contained the P5 and P7 adaptor sequences required for sequencing on the Illumina Hi-Seq 2000 platform. Furthermore, the forward primer carried a six-nucleotide barcode sequence between the loop- and the P7-sequence (modified from Scuoppo et al., 2012). Approximately $10x10^6$ 50-base-pair reads were acquired for each sample. shRNAs were identified by sequence alignment using the BWA algorithm (Li & Durbin, 2009). To determine shRNA distributions among different samples, raw read counts were normalized based on the size of each sequencing library by dividing each value to the number of total aligned reads for that sample and multiplying to $10^{\wedge 6}$. shRNA abundance ratios were calculated, for each shRNA, as the number of normalized reads in each tumor divided by the number of normalized reads at T0 (before injection). Normalized Fold Changes from three lymphomas/mouse were then averaged and log2 transformed. See Supplemental supplemental table S7 for the full list of primers.

**Cell cycle kinetics**

For BrdU pulse-chase experiment Eµ-*myc* lymphoma cells transduced with either empty vector (EV) or shPTCD3 #4 were grown in the presence of 33 µM BrdU for 15 minutes. At the end of BrdU incubation the medium was removed, cells were washed twice with PBS, supplemented with fresh medium and maintained in culture over the time-course. At 0, 2, 4, 6, 8, 10, 12, 14 and 24 hours, $3x10^6$ cells were collected, fixed in 70% Ethanol and kept at +4°C before staining. Staining was performed as described previously with minor modifications (Erba et al., 1999). For detection of BrdU incorporation into DNA, the fixed cells were washed with cold PBS and the DNA was denatured with 1 ml of 3N hydrochloric acid for 20 min at room temperature. DNA denaturation was stopped by adding 3 ml of 0.1M sodium tetraborate pH 8.5. After centrifugation, the pellet was washed once with 1 ml of PBS+1% BSA and incubated with a mouse monoclonal anti-BrdU antibody (Becton Dickinson, cat. #340649) diluted 1:5 in PBS+1% BSA and kept for 1 h at room temperature in the dark. The pellet was washed once with 1 ml of PBS+1% BSA before incubation with an allophycocyanin (APC)-conjugated donkey anti-mouse secondary antibody (Invitrogen, cat. #A-31571) diluted

1:100 in PBS+1%BSA for 1 h at room temperature in the dark. The cells were finally resuspended in 1 ml of a solution containing 2.5 µg/ml of propidium iodide (PI) in PBS and 40µg/ml RNAse A, and stained overnight at 4°C in the dark.

For DNA content analysis cells were fixed with 70% EtOH, resuspended in a solution containing 50 µg/ml of propidium iodide (PI) in PBS and 40µg/ml RNAse A and stained overnight at 4°C in the dark. Samples were acquired on a FACSCalibur flow cytometer (Becton Dickinson).

**Electron Microscopy**

After treatment of cells in suspension with 1 or 2.5 µM Tigecycline for 12, 24 and 48 hrs, cells were fixed with 1% glutaraldehyde for 1 hour and then with reduced OsO4 for 2 hours and thiocarbohydrazide as described in (Polishchuk et al., 1999) and (Cutrona et al., 2013) and embedded into Epon. Standard 60 nm sections with the thicknes fo 60 nm were examined udner the Tecnai 20 electron microscope and the severity of mitochondrial damages was evaluated blindly by two experts.

**Assessment of Tigecycline Activity in Mouse lymphomas models**

For *in vivo* treatment with tigecycline, $2x10^5$ Eµ-myc lymphoma cells (LY36 or LY35) were transplanted by tail-vein injection into 6-8 weeks old healthy syngeneic C57Bl/6 recipient mice. Mice were monitored daily for lymphoma development by peripheral lymph node palpation. Treatment with tigecycline was started when lymph-nodes became palpable and mice showed evident signs of disease. For the xenograft model of human Burkitt's lymphoma, $10^7$ Raji cells were transplanted subcutaneously in previously irradiated (3 Gray) nude mice (Hudson et al., 1998). Treatment with tigecycline started upon the appearance of measurable tumors. Tumor volumes was assessed from the start of the treatment every two days with a digital caliper. In both cases we used the following treatment scheme: Two rounds of treatment twice a day for 5 days, with two days off at each round, for a total of 14 days and 20 treatments. Time interval between the two injections was ~6 hours. Tigecycline was dissolved in saline solution (0.8% NaCl) at 100mg/kg and was freshly prepared from the dry powder at each injection.

For Blood analysis, 50µl of blood was collected by tail-vein bleeding and 10µl 0.5M EDTA was immediately added to prevent coagulation. Whole blood was analysed using the Hematological Analyzer (Beckman Dickinson). White Blood Cells (WBC) were isolated from spleen by mechanical disruption and filtration thruogh a 70µM nylon cell strainer. Following lysis of erythrocytes peripheral or splenic WBC were stained with an anti-B220 PE-cy7 antibody (#12-0452-82, eBiosciences) and analyzed by flow-cytometry on a FACSCalibur (BD biosciences).

## SUPPLEMENTARY REFERENCES


Dickins RA, Hemann MT, Zilfou JT, Simpson DR, Ibarra I, Hannon GJ, Lowe SW (2005) Probing tumor phenotypes using stable and regulated synthetic microRNA precursors. Nature genetics 37: 1289-95

Dimri GP, Lee X, Basile G, Acosta M, Scott G, Roskelley C, Medrano EE, Linskens M, Rubelj I, Pereira-Smith O, et al. (1995) A biomarker that identifies senescent human cells in culture and in aging skin in vivo. Proc Natl Acad Sci U S A 92: 9363-7

Erba E, Bergamaschi D, Ronzoni S, Faretta M, Taverna S, Bonfanti M, Catapano CV, Faircloth G, Jimeno J, D'Incalci M (1999) Mode of action of thiocoraline, a natural marine compound with anti-tumour activity. British journal of cancer 80: 971-80

Hudson WA, Li Q, Le C, Kersey JH (1998) Xenotransplantation of human lymphoid malignancies is optimized in mice with multiple immunologic defects. Leukemia 12: 2029-33

Li H, Durbin R (2009) Fast and accurate short read alignment with Burrows-Wheeler transform. Bioinformatics 25: 1754-60

Scuoppo C, Miething C, Lindqvist L, Reyes J, Ruse C, Appelmann I, Yoon S, Krasnitz A, Teruya-Feldstein J, Pappin D, Pelletier J, Lowe SW (2012) A tumour suppressor network relying on the polyamine-hypusine axis. Nature 487: 244-8

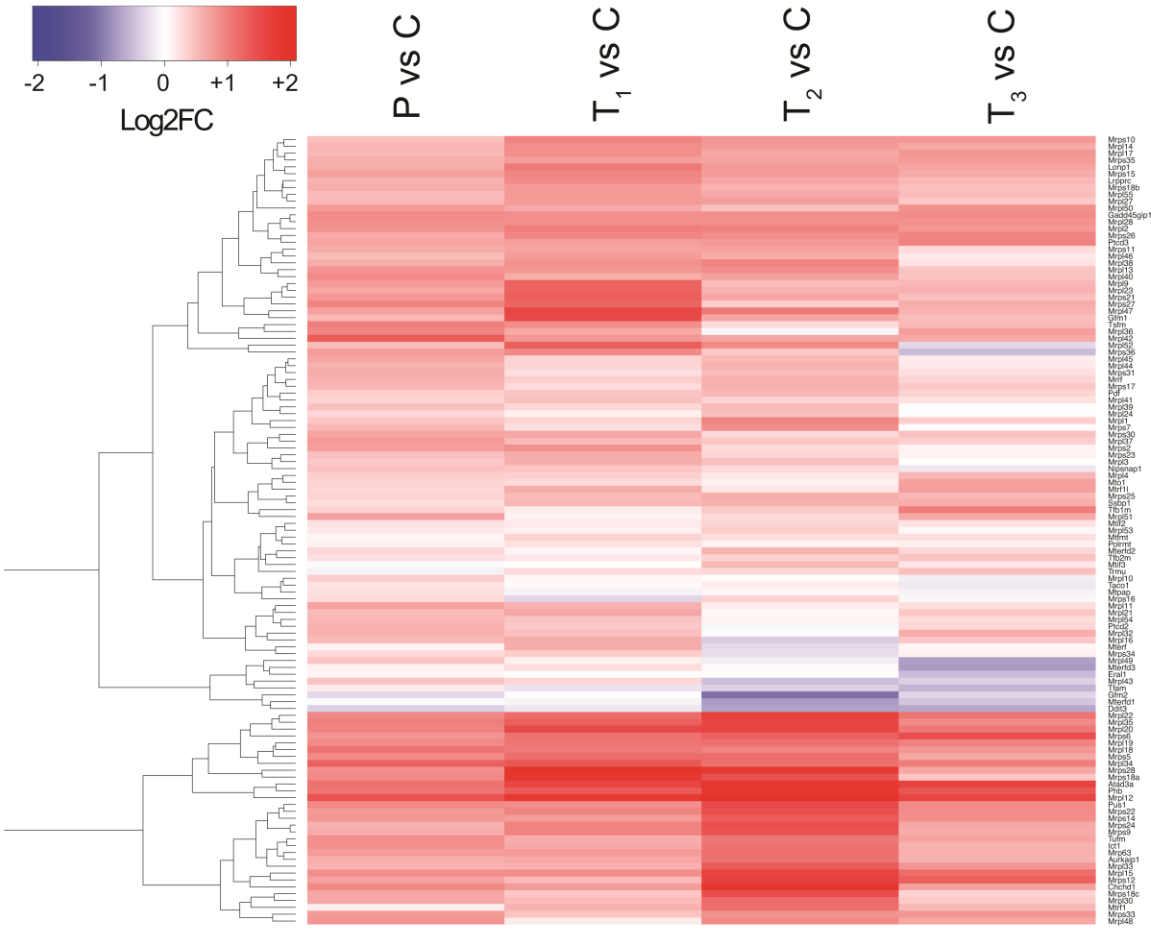


**Supplementary Figure S1. Genes linked to mitochondrial translation are coordinately up-regulated during Eμ-*myc* lymphomagenesis.**

Heatmap showing log2-transformed fold change values in Eμ-*myc* pre-tumoral (P) or tumor ($T_{1-3}$) cells versus non-transgenic control (C) B-cells for 115 different genes involved in transcription, processing and translation of the 13 mitochondrially-encoded RNAs. The list of genes was manually compiled based on functional annotation of genes from the NCBI's database Entrez-Gene.

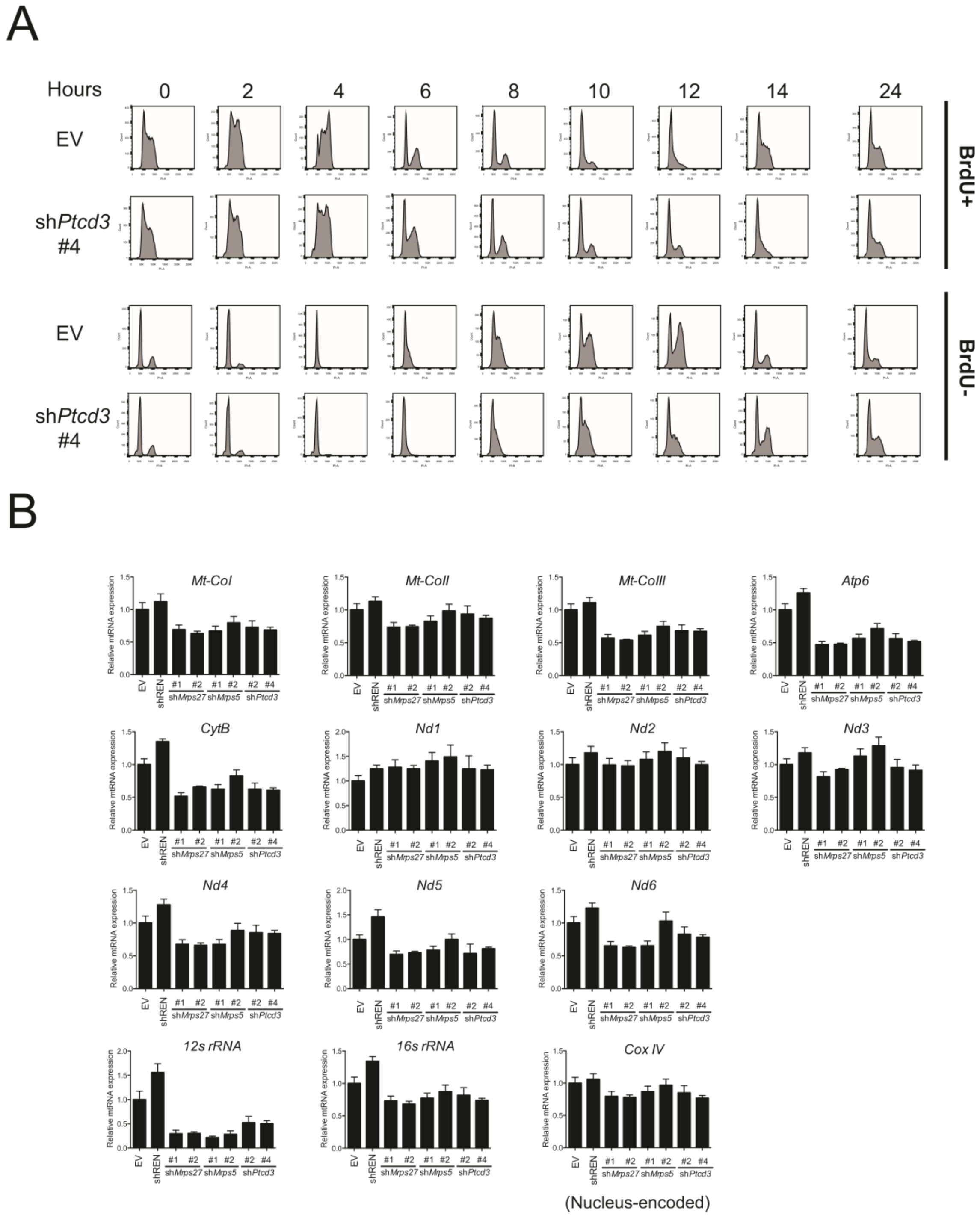


**Supplementary Figure S2. Knockdown of MRPs impairs the proliferation of Eμ-*myc* lymphomas.**

**A.** Bromodeoxyuridine (BrdU) pulse-chase analysis of Eμ-*myc* lymphoma cells infected with either an empty vector (EV) control or an shRNA against *Ptcd3* (sh #4). Following a 20 minute pulse with BrdU, time points were collected every two hours up to 14hrs and

then at 24hrs. DNA content histograms are shown separately for both BrdU+ and BrdU – populations, as indicated. **B.** The expression levels of 11/13 mitochondrion-encoded RNAs (mtRNAs), 12s and 16s mitochondrial ribosomal RNA (rRNA) and a nucleus-encoded messenger RNA (Cox IV) were evaluated by real-time qPCR 48hrs post-transduction. Transcript abundance is expressed as mean ± s.d. relative to EV control and normalized to *Rplp0*.

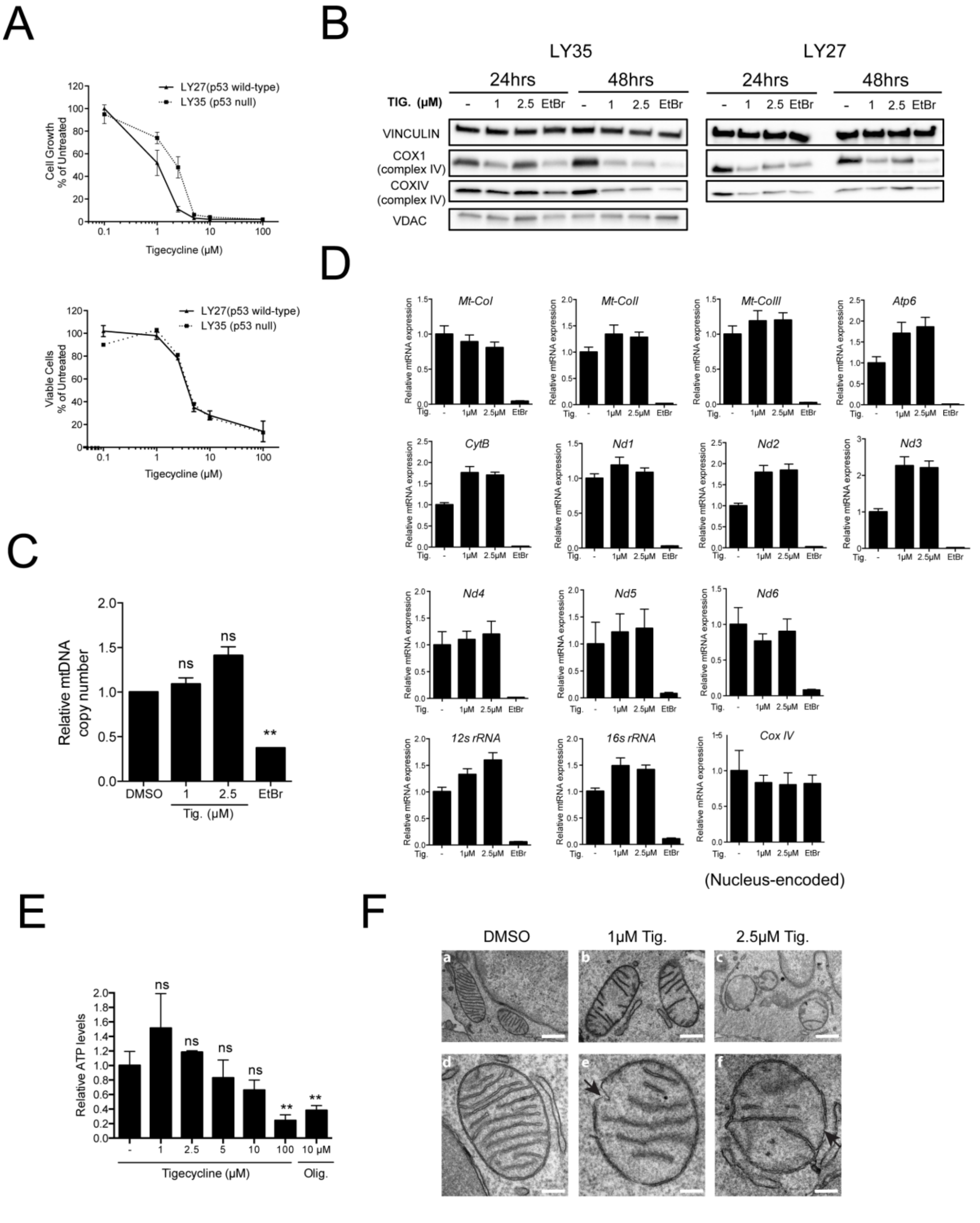


**Supplementary Figure S3. Effects of tigecyline on the growth of Eμ-*myc* lymphomas.**

**A.** Dose-response curve of two different Eμ-*myc* lymphomas (LY27 and LY35) treated with the indicated doses of Tigecycline for 48hrs. Data are shown as cell numbers (left panel) or viability (right panel), both as percentage of untreated cells (mock-treated with

DMSO). Cell number and viability were determined by trypan blue staining. B**.** Western blot analysis of ETC complex IV subunits COXI and COXIV on the lymphoma lines LY27 (the same as in Fig. 3C) and LY35. Cells were treated with the indicated concentrations of Tigecycline for 24 or 48hrs, as indicated. The loading controls are Vinculin and VDAC, as in Figure 2D. EtBr: as control, cells were treated with Ethidium Bromide, causing loss of mtDNA (see panel C). **C.** RT-PCR quantification of mtDNA, as in Figure 2A. **D.** The expression levels of 11/13 mtRNAs, 12s and 16s mitochondrial ribosomal RNA and a nucleus-encoded messenger RNA (*Cox IV*) were evaluated by real-time qPCR after 24hrs of treatment with the indicated doses of Tigecycline. Transcript abundance is expressed as mean ± s.d. relative to empty vector (EV) control and normalized to *Rplp0*. **E.** Eµ-*myc* lymphoma cells were treated with Tigecycline at the indicated doses for 6hrs. ATP levels were measured with the Cell Titer Glo assay on equal numbers of cells and are expressed relative to DMSO-treated cells (-). Olig.: oligomycin-treated cells were used as positive control. **F.** Electron microscopy pictures of Eµ-*myc* lymphoma cells treated with DMSO, 1µM or 2.5µM Tigecycline for 12hrs. DMSO-treated cells exhibited normal mitochondrial morphology (a, d). In cells treated with 1 µM Tigecycline for 12 hrs, a small percentage of mitochondria showed reduced number of cristae and/or perforation of the external mitochondrial membrane (b, e). Treatment with 2.5µM led to a more consistent reduction in number and lenght of cristae and damages of the external membrane (arrows) were observed at higher frequency (f). Scale bars: a (700 nm); b (300 nm); c (600 nm); d (200 nm ); e, f (150 nm).

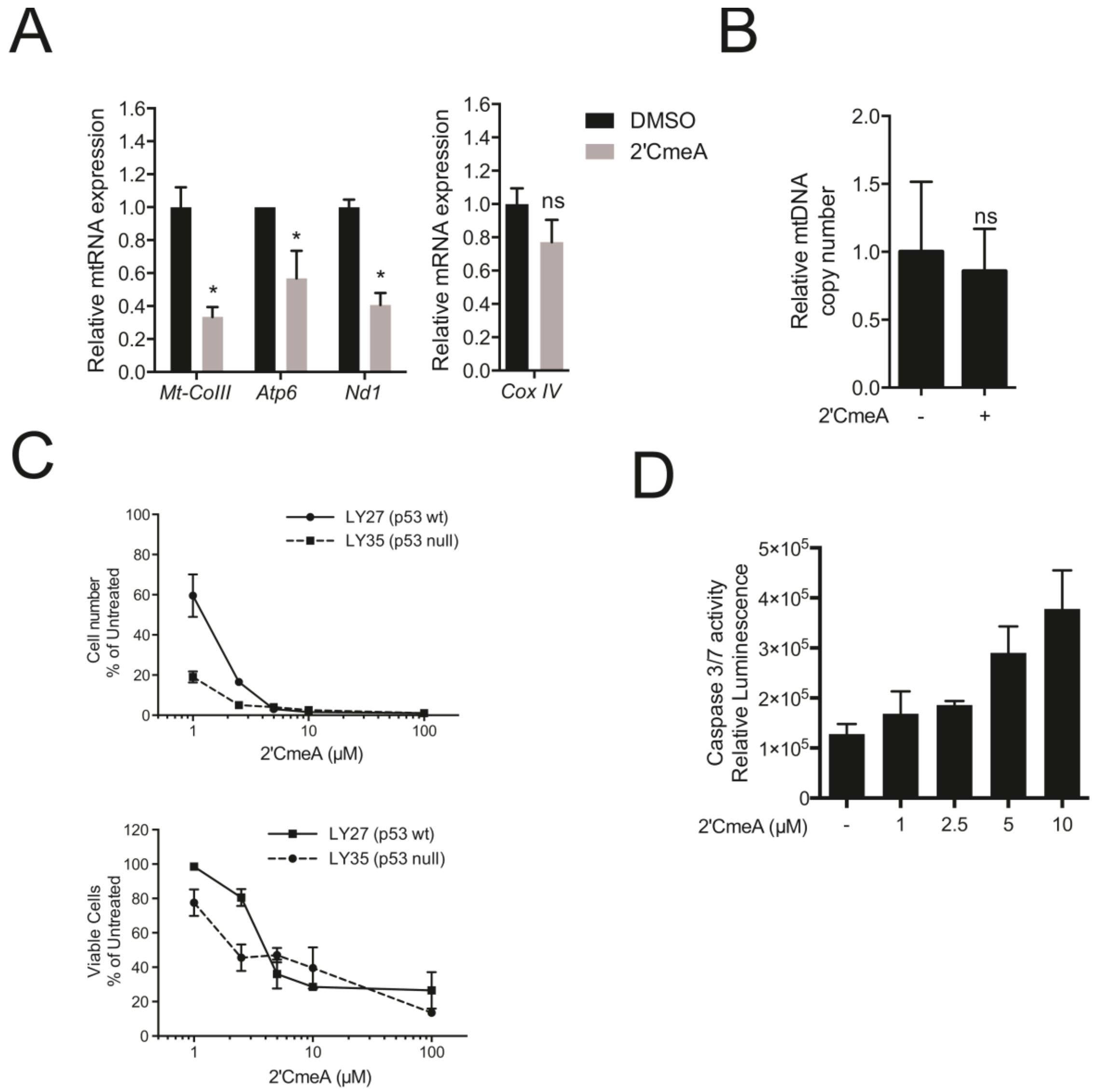


**Supplementary Figure S4. Effects of 2'-C-Methyladenosine on the growth of Eμ-*myc* lymphomas.**

**A.** *Cox-III, Atp6, Nd1* mtRNAs (left) and *Cox-IV* mRNA levels (right) were quantified by RT-PCR in Eμ-*myc* cells treated with 10μM of 2'-C-Methyladenosine (2'CmeA) for 6hrs relative to DMSO-treated samples. Data were normalized to *Rplp0* expression. **B.** RT-PCR quantification of mtDNA in cells treated with 10μM 2'CmeA for 24hrs, as in Figure 2A. **C.** Total cell numbers (top) and Cell viability (bottom) for two different Eμ-*myc* lymphomas (LY27 and LY35) treated with the indicated doses of 2'CmeA for 48hrs. Values are represented as in Figure S3A. **D.** Cleaved caspase 3/7 activity was determined after 6 hours of treatment with 2'CmeA, as in Figure 3E . All plotted values are the mean

± s.d. from three independent experiments. * p <0.05; ns, not significant as determined by Student's t test.

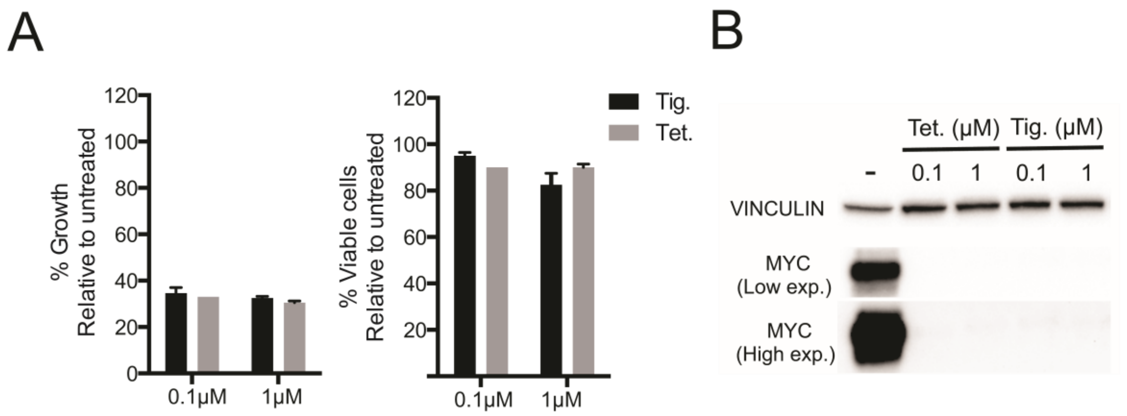


**Supplementary Figure S5. Tigecycline represses Myc expression in P493-6 cells**

**A.** Quantification of cell growth (left) or viability (right) in P493-6 cells treated with the indicated concentrations of Tigecycline or Tetracycline for 48hrs, relative to mock-treated cells. **B.** Western blot analysis of Myc expression in P493-6 cells following treatment with the indicated doses of either Tigecycline or Tetracycline for 48hrs. Vinculin was used as loading control. Two different exposure times are shown for the Myc immunoblot (high and low exp.).

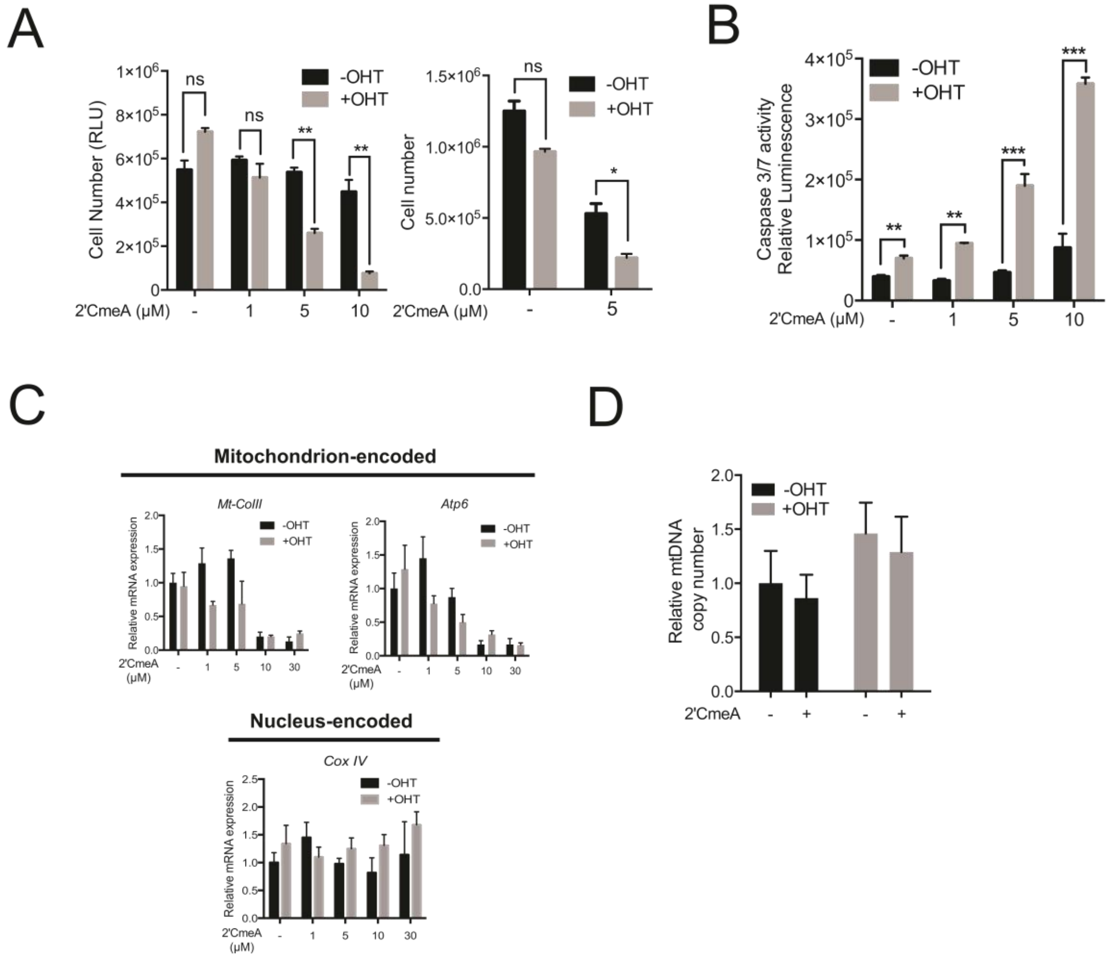


**Supplementary Figure S6. Synthetic lethality between Myc and Tigecycline in mouse mammary epithelial cells (MMECs).**

**A.** Relative numbers of R26-MER$^{T2}$ MMECs, as measured with cell titer glo (left panel) or trypan blue exclusion assay (right panel). Cells were treated with the indicated doses of 2'C-methyladenosine (2'CmeA) for 48 hours, with or without OHT. **B.** Caspase 3/7 activity was measured after 48 hours of treatment with 2'CmeA at the indicated doses, as in Fig 3F. The bars represent absolute luminescence signals in untreated untreated versus OHT-treated samples. **C.** *Mt-CoIII*, *Atp6* mtRNA and *Cox-IV* mRNA levels were quantified by RT-PCR in MMECs cells treated with the indicated doses of 2’CmeA for 24hrs with or without 20nM OHT. Data were normalized to *Rplp0* expression. **D.** RT-PCR quantification of mtDNA in MMECs treated with 5μM 2’CmeA for 24hrs, in the absence or presence of 20nM OHT. Data are normalized to nuclear DNA. All plotted

values are the mean ± s.d. from three independent experiments. * $p < 0.05$; ** $p < 0.01$; *** $p < 0.001$; ns, not significant as determined by Student's t test.

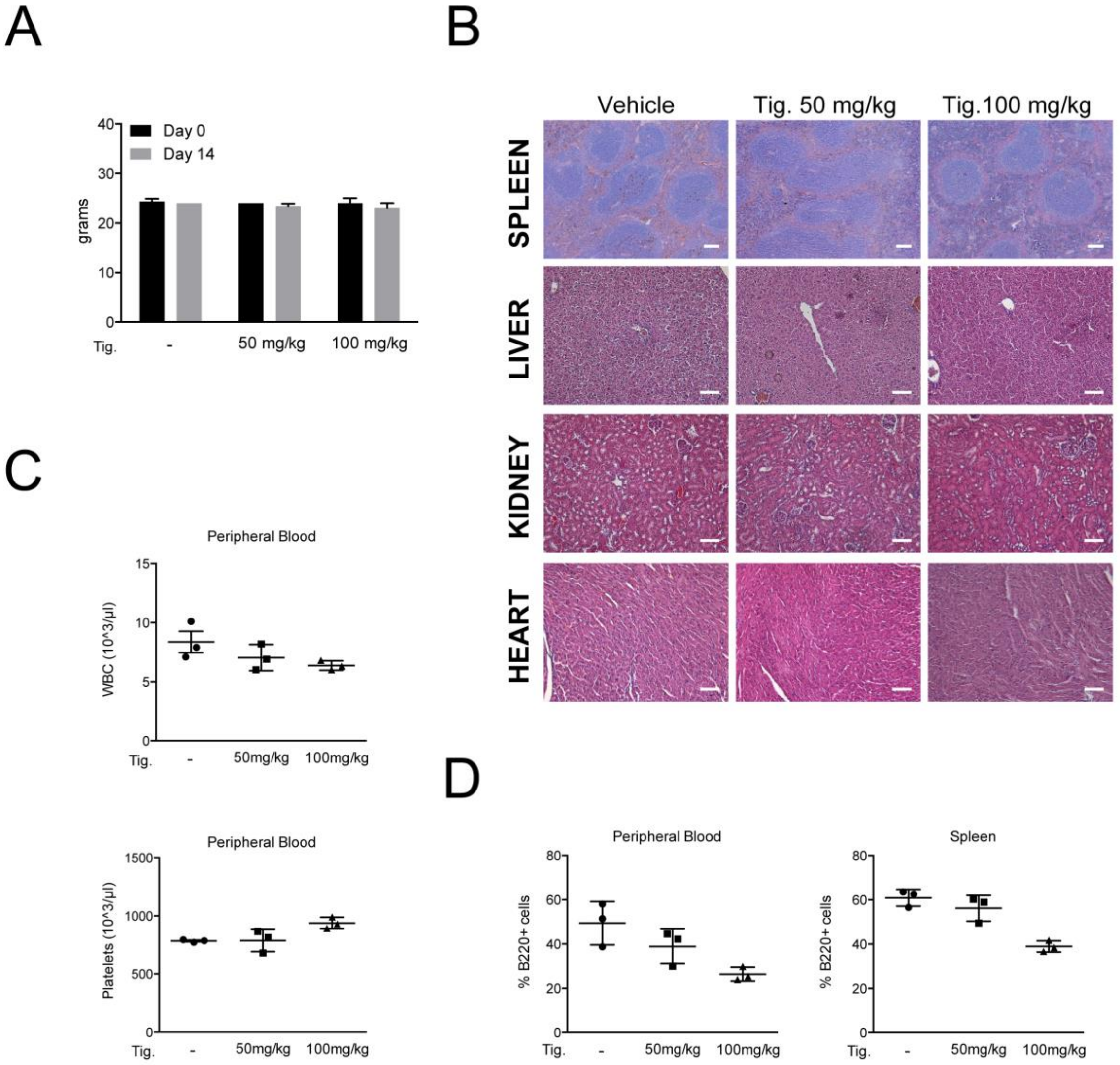


**Supplementary Figure S7. Toxicological evaluation of Tigecycline *in vivo*.**

**A.** Mouse weight was determined at the beginning (Day 0) and end (Day 14) of Tigecycline treatment. Results are shown as mean ± s.d. for three mice per each experimental group. **B.** Representative H&E images of spleen, liver, kidney and heart of mice treated bi-daily for 14 days with 50 or 100 mg/kg Tigecycline or saline solution (vehicle). Scale bar 100 µm (liver, kidney, heart), 200µm (spleen). **C.** Total number of white blood cells (WBC) and platelets (PLT) of mice treated as in A. **D.** Percentage of

B220+ cells in peripheral blood (left panel) and spleen (right panel) as determined by FACS staining with an anti-B220 antibody. In panels C. and D., results are shown as the mean ± s.d. from three mice per experimental condition; differences between treated and untreated samples were all below statistical significance.
